# A Minimally Invasive Transthoracic Injection Technique for Reproducible Intrapleural Delivery in Mice

**DOI:** 10.3390/mps8030055

**Published:** 2025-05-28

**Authors:** Sophie Rovers, Pooyeh Farahmand, Dana Liu, Louize Brants, Christophe Hermans, Dieter Peeters, Danielle McKinven, Jennifer Doig, Filip Lardon, Jan van Meerbeeck, Elly Marcq, Daniel J. Murphy, Evelien Smits

**Affiliations:** 1 Center for Oncological Research (CORE), University of Antwerp, 2000 Antwerpen, Belgiumevelien.smits@uantwerpen.be (E.S.); 2 School of Cancer Sciences, University of Glasgow, Glasgow G61 1BD, UK; pooyeh.farahmand@glasgow.ac.uk (P.F.); daniel.murphy@glasgow.ac.uk (D.J.M.); 3 Department of Pathology, Antwerp University Hospital, 2650 Edegem, Belgium; 4 Multidisciplinary Oncological Center Antwerp (MOCA), Antwerp University Hospital, 2650 Edegem, Belgium; 5 European Reference Network for Rare or Low Prevalence Lung Diseases—ERN-LUNG, 60596 Frankfurt am Main, Germany; 6 Laboratory of Dendritic Cell Biology and Cancer Immunotherapy, VIB Center for Inflammation Research, 9052 Gent, Belgium; elly.marcq@vub.be; 7 Brussels Center for Immunology, Vrije Universiteit Brussel, 1050 Ixelles, Belgium; 8 CRUK Scotland Institute, Glasgow G61 1BD, UK

**Keywords:** orthotopic models, intrapleural injection, pleural mesothelioma, transthoracic injection, minimally invasive methods, preclinical models

## Abstract

The development of standardised, reproducible preclinical models is essential for advancing pleural mesothelioma (PM) research. Here, we present a simple and reliable minimally invasive transthoracic intrapleural injection technique that could improve the efficiency of orthotopic PM model generation. By incorporating a simple needle sleeve to control the injection depth, this method eliminates the need for surgery or general anaesthesia, reducing technical complexity and animal stress while ensuring precise delivery into the pleural cavity. We demonstrate the effectiveness of this approach by achieving a 100% tumour engraftment rate following the injection of AE17 tumour cells. Additionally, this technique has been successfully used for asbestos fibre injection in mesothelioma models, highlighting its versatility. By providing a more accessible, standardised alternative to existing methods, this protocol improves the reliability of PM models and facilitates broader adoption by researchers, including those with limited experience in invasive procedures.

## 1. Introduction

Pleural mesothelioma (PM) is an aggressive malignancy affecting the pleura of the lungs, typically linked to asbestos exposure. Despite recent advances in treatment, particularly within immunotherapy, the prognosis for PM remains poor, highlighting the urgent need for further research to develop more effective therapies [1]. Preclinical models play a critical role in this effort, providing a platform for evaluating novel treatments before clinical translation. Among these, in vivo tumour models are essential for optimising therapeutic strategies and understanding disease progression.

Murine cancer models are invaluable tools for studying tumour biology and assessing potential therapies. Subcutaneous tumour models, in which tumour cells are implanted beneath the skin, are widely used due to their ease of implementation and straightforward tumour monitoring. However, these models fail to replicate the tumour’s native microenvironment, limiting their translational relevance. In contrast, orthotopic tumour models, where tumour cells are implanted in their tissue of origin, better recapitulate disease pathology, including interactions with the tumour microenvironment and host immune system. In immunocompetent mice, orthotopic models allow for a more physiologically relevant evaluation of treatment efficacy [2,3,4,5,6].

A range of in vivo PM models have been developed, each with distinct advantages and limitations. For instance, asbestos exposure can be used to induce PM in mice (or rats), but inhalation-based approaches are difficult to regulate and require long latency periods before tumour formation [7,8]. More commonly, PM models are generated by implanting patient-derived material or injecting tumour cells into the pleural cavity. Patient-derived orthotopic xenografts (PDOXs) have been established through surgical orthotopic implantation (SOI), which involves the direct intrapleural placement of tumour fragments [6,9,10]. Studies have shown that orthotopic models more accurately mimic PM disease progression compared to subcutaneous models [11,12], reinforcing their value in preclinical research.

However, generating orthotopic PM models presents significant challenges. The most commonly used approach involves the intrathoracic or intrapleural implantation of tumour material into athymic nude mice, which not only requires surgical expertise but also limits the study of immune-related mechanisms due to the host being immunodeficient [9,10,11,13]. Additionally, SOI methods, such as transdiaphragmatic implantation, necessitate general anaesthesia and surgery, increasing the technical complexity, procedural risk (e.g., pneumothorax), and inefficiency [14,15]. While some studies have attempted minimally invasive approaches to transthoracic or intrapleural injections, these techniques have generally been performed under general anaesthesia without direct visualisation or standardisation of the depth of injection, making them “blind” procedures with an inherent risk of off-target inoculation [16,17].

In this protocol, we describe a refinement of the minimally invasive transthoracic injection method, involving the addition of a needle sleeve to control the injection depth. This modification reduces technical difficulty, eliminates the need for surgery or anaesthesia—thereby minimising procedural stress on animals—and enhances reproducibility. Although this technique has been used in previous studies [12,18,19], it has never been thoroughly detailed. Here, we provide a comprehensive and standardised description of this approach, facilitating its broader adoption and improving the efficiency of orthotopic PM model generation.

## 2. Experimental Design

**Figure 1:**
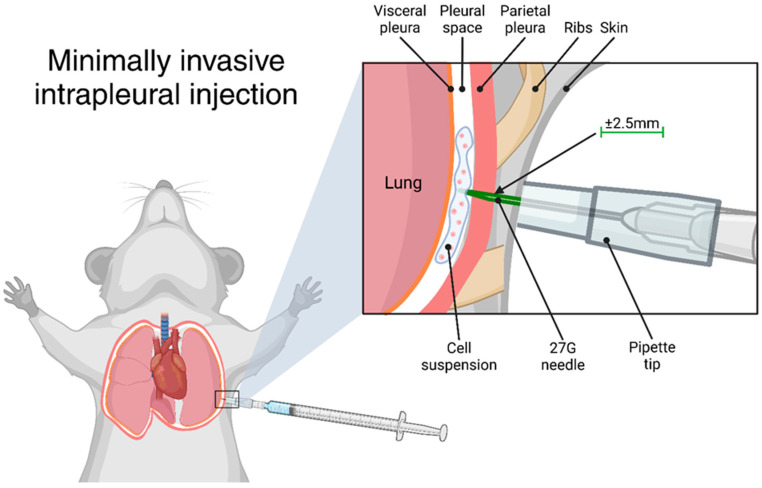
Graphical representation of the transthoracic intrapleural injection. Created using BioRender.com.

The protocol described here enables the minimally invasive transthoracic intrapleural injection of liquid agents in mice, offering a standardised and reproducible approach to preclinical studies of pleural disease. It eliminates the need for surgery or anaesthesia and is suitable for immunocompetent mice. The representative results described below illustrate the application of this injection technique and how its success can be verified immediately post-injection. While this method can be used for various experimental purposes, the results presented here serve as an example of successful injection outcomes, not as an optimized model of pleural mesothelioma. The protocol is adaptable to the injection of tumour cells, asbestos fibres [19], or other agents. A schematic of the experimental procedure is provided in Figure 1.

To validate the accuracy and reproducibility, we conducted a series of control and experimental studies. Trypan Blue was used as a training and validation control to confirm precise intrapleural delivery. In the experimental setup, AE17 mesothelioma cells were injected intrapleurally and mice were monitored over time, with weight tracking used as an indicator of physiological changes. From day 15 post-injection, two mice were sacrificed each day for the post-mortem macroscopic and histological analysis of tumour development in the pleural cavity, including the lungs, heart, diaphragm, and thymus.

### 2.1. Materials

- RPMI 1640, HEPES (ThermoFisher Scientific, Merelbeke, Belgium, Cat. Number 52400025)
- Fetal Bovine Serum (ThermoFisher Scientific, Cat. Number 10270–106)
- L-Glutamine (ThermoFisher Scientific, Cat. Number 25030024)
- TrypLE Express Enzyme (ThermoFisher Scientific, Cat. Number 12604–021)
- Dulbecco’s Phosphate Buffered Saline (ThermoFisher Scientific, Cat. Number 14190–144)
- Trypan Blue solution 0.4% (ThermoFisher Scientific, Cat. Number 15250–061)
- BD Plastipak™ 1 mL luer slip syringes, concentric (VWR, Leuven, Belgium, Cat. Number 613–5399)
- BD Microlance™ 3 needles, 27 G, 13 mm long (VWR, Cat. Number 613–3832)
- 200 µL pipette tips (Sarstedt, Antwerp, Belgium, Cat. Number 70.3030)
- Azpack™ Carbon Steel Razor Blades (Fisher Scientific, Merelbeke, Belgium, part of ThermoFisher Scientific, Cat. Number 11904325)
- Soft-Zellin-C alcohol cleansing pads

### 2.2. Limitations

The success and reproducibility of this intrapleural injection technique rely on several key methodological considerations. A major advantage of this protocol is that the use of a pipette tip sleeve standardises the depth of injection, making accurate intrapleural delivery much easier compared to freehand or “blind” techniques. However, proper needle length selection remains important, as minor variations in needle size or individual mouse anatomy could influence depth control. Ensuring that no more than 2.5 mm of the needle tip is exposed minimises the risk of accidental lung parenchyma penetration while maximising reproducibility.

Correct needle positioning and proper animal handling are essential for accurate intrapleural injection. The injection should be performed below the axilla to avoid the fat pad in the armpit, and the mouse must be firmly restrained to minimise movement during injection and keep the skin taut over the ribcage. Suboptimal restraint may result in misplacement of the injection, compromising tumour engraftment and experimental outcomes. While this technique eliminates the need for general anaesthesia, which can introduce variability due to anaesthesia-related stress, it does require confident and consistent handling. To improve accuracy, researchers may benefit from first practicing the injection technique using a dye, such as Trypan Blue, as shown in Figure 1. This allows for visual confirmation of correct deposition within the pleural cavity before proceeding with applications such as tumour cell inoculation.

While this technique offers a minimally invasive and highly efficient method for tumour cell inoculation, it is not universally suited to all experimental needs. For example, alternative methods such as SOI may be preferable for delivering solid tumour fragments to generate PDOX, where precise tumour placement and microenvironment preservation are critical for optimal growth. This protocol, by contrast, is designed exclusively for liquid-based injections, making it less suitable for applications requiring solid tissue engraftment. Additionally, while this technique has been successfully applied for asbestos fibre injection to model mesothelioma [19], its feasibility for repeated intrapleural drug delivery or immune cell injections requires further exploration.

Finally, although tumour progression (following intrapleural inoculation of tumour cells) can be assessed through weight tracking and post-mortem analysis, the method does not provide real-time confirmation of tumour establishment. Future studies may integrate non-invasive imaging techniques, such as bioluminescence or micro-CT, to further validate tumour development and refine monitoring approaches.

## 3. Procedure

Female C57BL6/J mice (6 weeks old) were obtained from Charles River Laboratories (Saint-Germain-Nuelles, France) and maintained under standard housing conditions at the animal facility of the University of Antwerp. The following protocol is in accordance with the University of Antwerp’s animal ethics guidelines (Ethical Committee for Animal Testing, University of Antwerp, Belgium, approval number ECD 2019-32) and with the European code for the care and use of laboratory animals.

### 3.1. Cell Suspension Preparation

1. AE17 mesothelioma cells are cultured in RPMI 1640 medium supplemented with 10% foetal bovine serum (FBS) and 2 mM of L-glutamine. After thawing, cells must be passaged at least twice but no more than five times before inoculation.
2. Detach cells from the culture flask TrypLE and wash them twice in sterile phosphate-buffered saline (PBS). Count the cells and resuspend them in an appropriate volume of sterile PBS to achieve the desired final concentration.
3. Assess cell viability using the trypan blue exclusion assay, ensuring a viability of >95% before injection.

### 3.2. Needle Preparation

1. Assemble a 0.3 mL or 1 mL syringe with a 27 G needle. Place a 100–200 µL (yellow) pipette tip over the needle, marking the pipette tip approximately 2.5 mm below the needle point.
2. Using a straight razor blade, cut the pipette tip at the marked location.
3. NOTE: Needle lengths may vary slightly. It is advisable to prepare multiple pipette tips of varying sizes to ensure a proper fit. Select the correct pipette tip length for each needle assembly.
4. Fill the syringe with sufficient cell suspension for 100 µL per mouse. It is recommended that a fresh needle is used for each animal.
5. Reassemble the cut pipette tip onto the needle, ensuring that no more than 2.5 mm of the needle tip is exposed. Secure the pipette tip in place with autoclave tape if necessary.

The syringe is now ready for use.

### 3.3. Transthoracic Injection

1. Restrain the mouse by firmly scruffing it. Ensure that its front limb (right for right-handed handlers, left for left-handed handlers) is lifted and immobilised. The skin should be pulled taut over the ribcage.
2. Disinfect the injection site with an ethanol wipe and part the fur to expose the skin. Identify the intercostal space below the axilla (avoiding the fat pad in the armpit).
3. Insert the needle at the selected injection site with the bevel facing downward. Ensure the pipette tip is flush against the skin, applying gentle pressure.
4. Slowly inject 100 µL of the cell suspension. After injection, release the mouse and observe it for a few minutes to monitor for signs of pneumothorax (e.g., laboured breathing, signs of distress). If pneumothorax occurs, euthanise the mouse immediately.

### 3.4. In Vivo Monitoring

1. Monitor body weight and general health throughout the experiment. See Expected Results for tumour kinetics.
2. Potential clinical symptoms include respiratory distress (e.g., shortness of breath), weight gain due to fluid accumulation (ascites), lethargy, and sudden weight loss. Mice should be euthanised if at least two of these symptoms are observed.

### 3.5. Pleural Fluid Collection

1. To collect pleural fluid, the animal must lay flat on its back after euthanasia and four paws should be pinned to the work surface area.
2. Make a small transverse incision, approximately 1 cm, on the abdomen using a sharp pair of scissors followed by a vertical anterior incision along the midline up to the top of the mediastinum. Open the skin from both sides. Then perform similar transverse incision in the peritoneal wall. The anterior incision of the peritoneal wall should only be large enough to expose the liver/stomach. It is very important to keep the pleural cavity intact at this stage.
3. Using a pair of tweezers, carefully move back the liver to expose the entire diaphragm and visualise the effusion.
4. Carefully insert a 25 G needle into the pleural space through the diaphragm, where the effusion has accumulated. Gently collect the fluid in a syringe (the needle might need to be repositioned at times). The fluid can then be transferred into an appropriate tube and stored accordingly for further analysis.
5. OPTIONAL STEP: If little or no spontaneous effusion is present, the user may wish to perform a lavage, which can be performed with up to 1 mL of balanced salt solution or standard tissue culture media. Perform steps 1 to 4 as described above by injecting the desired solution into the closed chest cavity through the diaphragm prior to withdrawing the fluid through the same injection site.

### 3.6. Tumour Collection

1. After collecting pleural fluid, circumferentially dissect the diaphragm to detach it. Carefully inspect the diaphragm and collect any tumours growing on its surface.
2. Perform a median sternotomy by cutting along both sides of the sternum and removing it. This exposes the heart, lungs, and thymus. Inspect the thoracic cavity thoroughly for tumours, which may be present on the chest wall, lungs, heart, thymus, or diaphragm. Tumours typically appear light in colour, smooth or slightly nodular, and firm.
3. Excise all visible tumours and process them for embedding and further histological analysis as required.

## 4. Expected Results

### 4.1. Validation of Injection Accuracy

For researchers unfamiliar with transthoracic intrapleural injections, one of the key challenges is confirming whether injection was performed correctly. Because injected tumour cells are not immediately visible, it can be difficult—especially for inexperienced researchers—to determine if the needle successfully delivered its contents into the pleural cavity rather than an unintended site. To address this, we demonstrate a simple yet effective method using Trypan Blue, which allows for the immediate verification of injection accuracy.

**Figure 2:**
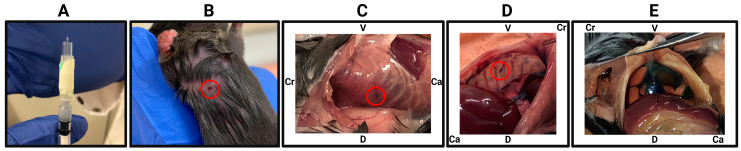
Immediate verification of intrapleural injection accuracy using Trypan Blue. (**A**) Injection setup, showing the 27 G needle with an attached sleeve (cut 200 µL pipette tip) exposing approx. 2.5 mm of the needle tip to control penetration depth. (**B**) Injection site marked on a restrained mouse, target area indicated by red circle. (**C**) Post-mortem examination immediately following injection, showing the needle entry point between the ribs (red circle). (**D**) Internal view of the thoracic cavity showing the needle entry point between the ribs (red circle). (**E**) Trypan Blue visible through the intact diaphragm, verifying successful pleural cavity delivery. V, ventral; D, dorsal; Cr, cranial; Ca, caudal.

To demonstrate this approach, we injected mice with Trypan Blue and immediately euthanised the mice to perform a post-mortem examination. Successful injections were confirmed by the presence of dye at the expected thoracic entry site and within the pleural cavity (Figure 2). This method allows researchers to refine their technique and ensure consistent, accurate delivery before proceeding with experimental injections.

### 4.2. Post-Injection Weight Monitoring Reveals Variable Physiological Responses

**Figure 3:**
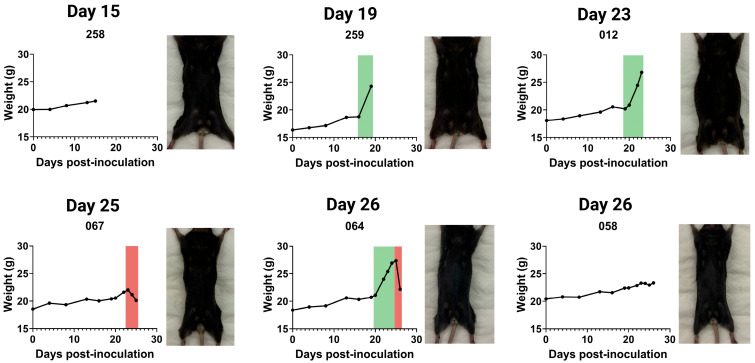
Weight progression and physical appearance of mice following intrapleural injection of AE17 murine mesothelioma cells. Mice received an intrapleural injection of 0.1 × 10^6^ AE17 murine mesothelioma cells and were euthanised daily from day 15 post-inoculation. For each day, we present weight curves and the corresponding post-mortem images of euthanised mice (also see Figure A1). Weight progression varied between individual animals, with some mice exhibiting significant weight gain due to fluid accumulation (ascites, indicated in green), while others maintained stable weight or experienced rapid weight loss (red).

**Figure A1:**
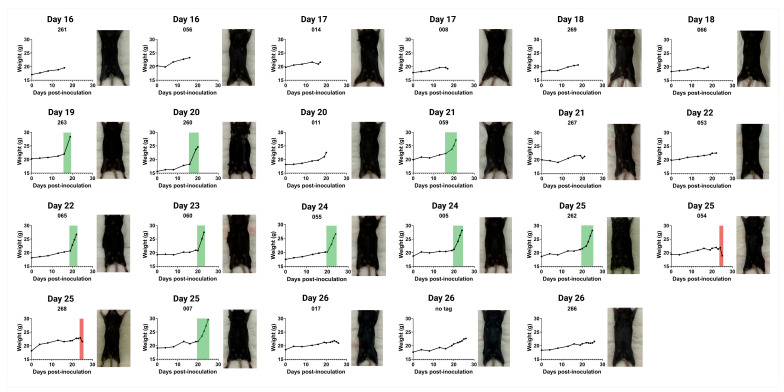
Weight progression and physical appearance of mice following intrapleural injection of AE17 murine mesothelioma cells. Mice received an intrapleural injection of 0.1 × 10^6^ AE17 murine mesothelioma cells and were euthanised daily from day 15 post-inoculation. For each day, we present weight curves and corresponding post-mortem images of euthanised mice. Weight progression varied between individual animals, with some mice exhibiting significant weight gain due to fluid accumulation (ascites, indicated in green), while others maintained stable weight or experienced rapid weight loss (red).

To illustrate the potential applications of this injection technique, we performed intrapleural injections of AE17 murine mesothelioma cells (0.1 × 106 cells in 100 µL) and monitored physiological changes over time. Mice were weighed daily, and from day 15 post-inoculation, two mice were euthanised per day for post-mortem analysis. On days 25 and 26, five mice were euthanised due to reaching humane endpoints (Figure 3 and Figure A1).

Weight tracking revealed three distinct patterns: (1) sudden or gradual weight gain, often associated with ascites development; (2) stable weight, with tumour presence confirmed post-mortem; and (3) sudden weight loss, frequently linked to systemic disease progression. Figure 3 presents the weight curves for a few mice illustrating these patterns, accompanied by corresponding post-mortem images. Mice with substantial weight gain displayed abdominal distension and pronounced fluid retention, while those with significant weight loss appeared visibly emaciated.

These observations highlight the variability in disease progression following intrapleural tumour cell injection. While weight monitoring provides a useful non-invasive indicator of disease burden, it is not always directly proportional to tumour growth. Thus, complementary assessments, including imaging or histopathological analysis, may be required for a comprehensive evaluation of tumour progression.

### 4.3. Macroscopic Examination Confirms Tumour Development in Injected Mice

**Figure 4:**
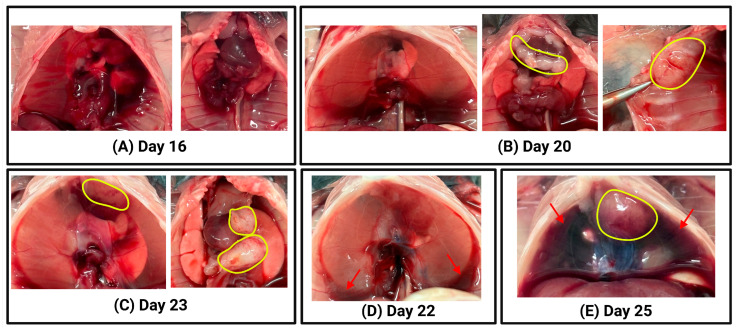
Macroscopic evaluation of thoracic tumour progression following intrapleural injection of AE17 mesothelioma cells. For each euthanised animal, photographs were taken during the post-mortem examination to assess tumour burden and pleural fluid accumulation. Representative examples illustrate different stages of tumour progression and fluid build-up. (**A**) Left: intact thoracic cavity with diaphragm; no visible tumours. Right: exposed thoracic cavity showing lungs, heart, thymus; no visible tumours. (**B**) Left: intact thoracic cavity with diaphragm; no visible tumours. Middle: exposed thoracic cavity with lungs, heart, thymus; visible tumours outlined in yellow. Right: Close-up of tumour (yellow) on the chest wall. (**C**) Left: intact thoracic cavity with diaphragm; visible tumour (yellow) inside pleural cavity. Right: exposed thoracic cavity with lungs, heart, thymus, and multiple visible tumours (yellow). (**D**) Intact thoracic cavity with diaphragm; no visible tumours. A small amount of pleural fluid is observed at the bottom of pleural cavity (red arrows). (**E**) Intact thoracic cavity with diaphragm; a tumour is visible inside the pleural cavity (yellow). A large volume of pleural fluid is present in the pleural cavity (red arrows).

To confirm successful tumour inoculation using the intrapleural injection technique, post-mortem macroscopic evaluation was performed on euthanised mice at various time points following AE17 mesothelioma cell injection. Figure 4 shows representative images illustrating the range of tumour burden and pleural fluid accumulation that were observed. These images serve as examples of the expected outcomes following successful intrapleural tumour cell injection.

**Figure 5:**
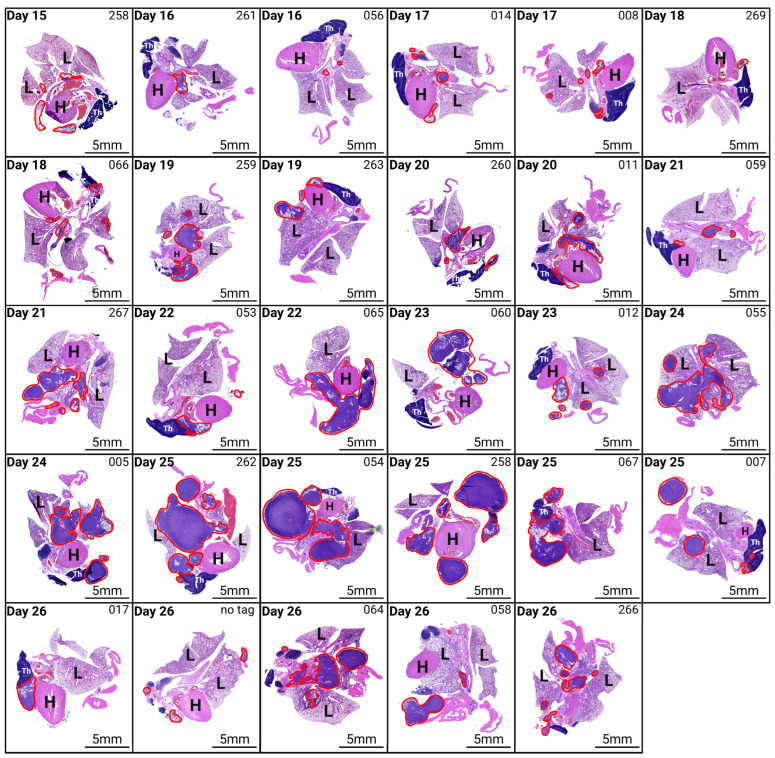
Histological analysis of tumour progression following intrapleural injection of AE17 mesothelioma cells. Haematoxylin and eosin (H&E) staining of lung, heart, thymus, diaphragm, and tumour tissue from mice euthanised at different time points post-inoculation. Mice were euthanised daily from day 15 post-inoculation. The figure displays representative histological images from different time points, showing tumour presence and progression over time. Tumour tissue is outlined in red. Magnification 10×. H, heart; L, lung; Th, thymus.

In some mice, especially at early time points (Days 15 to 18), no macroscopically visible tumours were detected upon inspection of the thoracic cavity (Figure 4A), although tumour presence was later confirmed microscopically (Figure 5), resulting in a 100% tumour engraftment rate. In others, tumour growth was evident on the chest wall, diaphragm, or within the pleural cavity (Figure 4B,C). The extent of tumour burden varied, with some mice presenting with discrete tumour nodules (Figure 4B) and others exhibiting more widespread tumour growth (Figure 4C). In addition to tumour formation, pleural fluid accumulation was observed in some animals, ranging from small amounts of fluid at the base of the pleural cavity (Figure 4D) to large volumes of pleural effusion (Figure 4E).

These findings confirm that the intrapleural injection method enables reliable tumour establishment in the pleural cavity. The variation in tumour burden and pleural fluid accumulation reflects the expected heterogeneity of tumour progression, further demonstrating the feasibility of this technique for preclinical mesothelioma studies.

### 4.4. Histological Analysis Demonstrates Progressive Tumour Growth

To further characterise tumour progression following the intrapleural injection of AE17 mesothelioma cells, histological analysis was performed on the lung, heart, thymus, diaphragm, and tumour tissue of euthanised mice (Figure 5). H&E staining revealed an increase in tumour burden over time, though with inter-individual variability.

From day 15 to day 18 post-inoculation, tumour presence was limited, often appearing as small tumour nodules within the pleural cavity. As the disease progressed, tumour infiltration became more extensive, with larger tumour masses adhering to and starting to invade the pleural surfaces, diaphragm, and pericardium. In some mice, tumour cells also appeared to be in the early stages of invading lymph nodes. By later time points, some mice exhibited widespread tumour dissemination throughout the thoracic cavity, with large tumours adhering to the lungs, heart, and thymus. However, not all mice followed the same pattern, emphasizing the heterogeneity of tumour growth even with the same inoculation technique.

These results demonstrate that successful intrapleural injection leads to tumour establishment and progression, making this technique a valuable tool for modelling pleural mesothelioma. However, the variability in tumour burden highlights the need for careful monitoring and standardised experimental conditions to further improve reproducibility.

## 5. Discussion

The development of reliable and reproducible preclinical models is essential for advancing PM research. In this study, we present a refined, minimally invasive intrapleural injection technique that addresses several limitations of traditional methods, offering a standardised approach with increased reproducibility. Our results demonstrate the technique’s effectiveness in tumour inoculation, achieving a 100% engraftment rate following AE17 cell injection, while also emphasising its versatility in facilitating other applications, such as asbestos fibre injection for mesothelioma modelling [19].

A key advantage of this method is its simplicity and the elimination of general anaesthesia and complex surgical procedures. By using a pipette tip sleeve to control the injection depth, our approach reduces the technical complexity associated with other methods, such as transdiaphragmatic injections [14,15,16], which require greater expertise and carry higher risks. The straightforward nature of the technique not only makes it more accessible to researchers with varying levels of experience but also enhances animal welfare by minimising stress and procedural risk.

Previous studies have demonstrated the variability and technical challenges associated with orthotopic mesothelioma models. For example, patient-derived xenograft (PDX) models generated through the subcutaneous implantation of PM tumour fragments reported engraftment rates as low as 40% [20]. In another study, Boehle et al. reported engraftment rates of 80–100% for various lung cancer cell lines using a transdiaphragmatic intrapleural injection, but this came at the cost of a procedure-related mortality rate of 11% [21]. Xu et al. directly compared two orthotopic approaches using A549-Luc2 human lung carcinoma cells and found that a transdiaphragmatic surgical approach had only 50% engraftment compared to 100% with a minimally invasive transthoracic injection. Moreover, the surgical procedure required up to 33 min per animal while the minimally invasive method required just 6–10 min [14]. These results demonstrate that this approach is compatible with human-derived cell lines and support its utility in cross-species applications.

In contrast, the technique described in this protocol achieved a 100% engraftment rate without any procedure-related mortality. The procedure duration was significantly shorter (approximately 2 min per mouse), and no anaesthesia or surgery was required. Although some variability in tumour kinetics and clinical symptoms was observed, as is typical for orthotopic models, these results underscore the robustness and reproducibility of this minimally invasive method. This work builds on previous efforts to standardise intrapleural injection techniques, such as the model described by Digifico et al., who also reported 100% engraftment using a minimally invasive transthoracic approach [12]. Notably, they validated this approach using three murine mesothelioma cell lines representing the epithelioid, sarcomatoid, and biphasic subtypes, demonstrating its suitability across histological variants. However, their method involved general anaesthesia and a small surgical incision, whereas our protocol eliminates both, further reducing technical complexity and animal burden. Additionally, the use of Trypan Blue as a verification tool provides an accessible training step, enabling researchers to confirm correct intrapleural delivery before proceeding with experimental inoculations. This aspect is particularly valuable for ensuring consistency across different laboratories and levels of expertise.

Beyond tumour inoculation, this technique holds promise for a variety of future applications in PM research. For instance, it could be adapted for intrapleural drug delivery [22] or cellular immunotherapy [23]. Moreover, its minimal technical requirements make it an excellent candidate for adoption in labs with limited access to complex equipment or high-level surgical expertise.

Despite these advantages, certain limitations must be considered. As discussed, this method requires proper animal restraint, and minor anatomical variations between mice may still introduce variability in injection success. Additionally, while weight tracking provided a useful indicator of tumour kinetics, non-invasive imaging techniques such as bioluminescence or micro-CT could further improve the monitoring of tumour establishment and progression. We recognise that this study did not include standardised quantitative assessments of tumour burden (e.g., tumour weight or digital image analysis), as the primary goal was to validate injection accuracy rather than to evaluate disease progression in detail. Future studies applying this technique should consider incorporating such quantitative endpoints to support reproducibility and enable more direct comparisons across laboratories.

In conclusion, this minimally invasive transthoracic intrapleural injection technique offers a valuable tool for preclinical PM research. By improving accessibility and reducing technical barriers, this method has the potential to facilitate broader adoption in preclinical research and contribute to the development of novel therapeutic strategies for PM.

## Data Availability

The raw data supporting the conclusions of this article will be made available by the authors on request.

## Abbreviations

The following abbreviations are used in this manuscript:

FBS	Foetal bovine serum
H&E	Haematoxylin and eosin
PBS	Phosphate-buffered saline
PDOX	Patient-derived orthotopic xenograft
PM	Pleural mesothelioma
SOI	Surgical orthotopic implantation

## References

[1] Baas P., Scherpereel A., Nowak A.K., Fujimoto N., Peters S., Tsao A.S., Mansfield A.S., Popat S., Jahan T., Antonia S. (2021). First-line nivolumab plus ipilimumab in unresectable malignant pleural mesothelioma (CheckMate 743): A multicentre, randomised, open-label, phase 3 trial. Lancet.

[2] Gengenbacher N., Singhal M., Augustin H.G. (2017). Preclinical mouse solid tumour models: Status quo, challenges and perspectives. Nat. Rev. Cancer.

[3] Stribbling S.M., Beach C., Ryan A.J. (2024). Orthotopic and metastatic tumour models in preclinical cancer research. Pharmacol. Ther..

[4] Day C.P., Merlino G., Van Dyke T. (2015). Preclinical mouse cancer models: A maze of opportunities and challenges. Cell.

[5] Stribbling S.M., Ryan A.J. (2022). The cell-line-derived subcutaneous tumor model in preclinical cancer research. Nat. Protoc..

[6] Lwin T.M., Hoffman R.M., Bouvet M. (2018). Advantages of patient-derived orthotopic mouse models and genetic reporters for developing fluorescence-guided surgery. J. Surg. Oncol..

[7] Seastedt K.P., Pruett N., Hoang C.D. (2021). Mouse models for mesothelioma drug discovery and development. Expert Opin. Drug Discov..

[8] Testa J.R., Berns A. (2020). Preclinical Models of Malignant Mesothelioma. Front. Oncol..

[9] Astoul P., Wang X., Colt H., Boutin C., Hoffman R. (1996). A patient-like human malignant pleural mesothelioma nude-mouse model. Oncol. Rep..

[10] Colt H.G., Astoul P., Wang X., Yi E.S., Boutin C., Hoffman R.M. (1996). Clinical course of human epithelial-type malignant pleural mesothelioma replicated in an orthotopic-transplant nude mouse model. Anticancer Res..

[11] Nakataki E., Yano S., Matsumori Y., Goto H., Kakiuchi S., Muguruma H., Bando Y., Uehara H., Hamada H., Kito K. (2006). Novel orthotopic implantation model of human malignant pleural mesothelioma (EHMES-10 cells) highly expressing vascular endothelial growth factor and its receptor. Cancer Sci..

[12] Digifico E., Erreni M., Colombo F.S., Recordati C., Migliore R., Frapolli R., D’Incalci M., Belgiovine C., Allavena P. (2020). Optimization of a Luciferase-Expressing Non-Invasive Intrapleural Model of Malignant Mesothelioma in Immunocompetent Mice. Cancers.

[13] Colin D.J., Cottet-Dumoulin D., Faivre A., Germain S., Triponez F., Serre-Beinier V. (2018). Experimental Model of Human Malignant Mesothelioma in Athymic Mice. Int. J. Mol. Sci..

[14] Xu J.J., Lucero M.Y., Herndon N.L., Lee M.C., Chan J. (2023). Comparison of a Minimally Invasive Transthoracic Approach and a Surgical Method For Intrapleural Injection of Tumor Cells in Mice. Comp. Med..

[15] Servais E.L., Colovos C., Kachala S.S., Adusumilli P.S. (2011). Pre-clinical mouse models of primary and metastatic pleural cancers of the lung and breast and the use of bioluminescent imaging to monitor pleural tumor burden. Curr. Protoc. Pharmacol..

[16] Vandeweerd J.M., Hontoir F., De Knoop A., De Swert K., Nicaise C. (2018). Retrograde Neuroanatomical Tracing of Phrenic Motor Neurons in Mice. J. Vis. Exp..

[17] Mantilla C.B., Zhan W.Z., Sieck G.C. (2009). Retrograde labeling of phrenic motoneurons by intrapleural injection. J. Neurosci. Methods.

[18] Murphy F.A., Poland C.A., Duffin R., Al-Jamal K.T., Ali-Boucetta H., Nunes A., Byrne F., Prina-Mello A., Volkov Y., Li S. (2011). Length-dependent retention of carbon nanotubes in the pleural space of mice initiates sustained inflammation and progressive fibrosis on the parietal pleura. Am. J. Pathol..

[19] Farahmand P., Gyuraszova K., Rooney C., Raffo-Iraolagoitia X.L., Jayasekera G., Hedley A., Johnson E., Chernova T., Malviya G., Hall H. (2023). Asbestos accelerates disease onset in a genetic model of malignant pleural mesothelioma. Front. Toxicol..

[20] Wu L., Allo G., John T., Li M., Tagawa T., Opitz I., Anraku M., Yun Z., Pintilie M., Pitcher B. (2017). Patient-Derived Xenograft Establishment from Human Malignant Pleural Mesothelioma. Clin. Cancer Res..

[21] Boehle A.S., Dohrmann P., Leuschner I., Kalthoff H., Henne-Bruns D. (2000). An improved orthotopic xenotransplant procedure for human lung cancer in SCID bg mice. Ann. Thorac. Surg..

[22] Li X., Wu G., Chen C., Zhao Y., Zhu S., Song X., Yin J., Lv T., Song Y. (2021). Intrapleural Injection of Anti-PD1 Antibody: A Novel Management of Malignant Pleural Effusion. Front. Immunol..

[23] Adusumilli P.S., Cherkassky L., Villena-Vargas J., Colovos C., Servais E., Plotkin J., Jones D.R., Sadelain M. (2014). Regional delivery of mesothelin-targeted CAR T cell therapy generates potent and long-lasting CD4-dependent tumor immunity. Sci. Transl. Med..

